# Forecasting Inequalities in Survival to Retirement Age by Socioeconomic Status in Denmark and Sweden

**DOI:** 10.1007/s10680-024-09704-8

**Published:** 2024-05-24

**Authors:** Cosmo Strozza, Marie-Pier Bergeron-Boucher, Julia Callaway, Sven Drefahl

**Affiliations:** 1 Interdisciplinary Centre on Population Dynamics, University of Southern Denmark, Odense, Denmark; 2 Demography Unit, Department of Sociology, Stockholm University, Stockholm, Sweden

**Keywords:** Income, Life expectancy, Lifespan inequality, Modal age at death, Pension policy, Social inequalities

## Abstract

In Denmark and Sweden, statutory retirement age is indexed to life expectancy to account for mortality improvements in their populations. However, mortality improvements have not been uniform across different sub-populations. Notably, in both countries, individuals of lower socioeconomic status (SES) have experienced slower mortality improvements. As a result, a uniform rise in the statutory retirement age could disproportionally affect these low-SES groups and may unintentionally lead to a reverse redistribution effect, shifting benefits from short-lived low-SES individuals to long-lived high-SES individuals. The aim of this study is twofold: to quantify and contextualise mortality inequalities by SES in Denmark and Sweden, and to assess how indexing retirement age will affect future survival to retirement age by SES in these countries. We used Danish and Swedish registry data (1988–2019), to aggregate individuals aged 50 + based on their demographic characteristics and SES. We computed period life tables by year, sex, and SES to estimate the difference in survival across different SES groups. We then forecast mortality across SES groups to assess how indexing retirement age will affect survival inequalities to retirement age, using two forecasting models—the Mode model and the Li-Lee model. Mortality inequalities are comparable in Denmark and Sweden, even though the latter generally has higher survival. We also find that indexing retirement age to life expectancy will have two main consequences: it will reduce the probability of reaching retirement for all SES groups, particularly those of low SES, and time spent in retirement will be reduced, particularly for those of high SES.

## Introduction

Countries with the highest life expectancies are experiencing unprecedented population ageing. More people in recent cohorts are reaching statutory retirement age than in previous ones, and when they retire, they are living longer (Burger et al., 2012; Vaupel et al., 2021). Population ageing poses unique challenges to the financial sustainability of pension systems (Sanderson & Scherbov, 2010, 2015). To rectify this, both Denmark and Sweden, as well as several other European countries, have implemented policies that index statutory retirement age to life expectancy. In other words, as life expectancy increases, so does retirement age.

When indexing life expectancy, calculations are performed at the national level and do not account for mortality differentials across sub-groups within the same country. Nevertheless, a social gradient in mortality has been observed in most countries with high life expectancies, including those with higher national incomes, social transfers, and healthcare expenditures, and among both men and women. These socioeconomic inequalities have persisted, and, in many cases, increased, over time (Clouston et al., 2016; Mackenbach et al., 2017). The lack of consideration for these differentials in the indexing process may inadvertently create a misalignment between the intended goal of pension systems to redistribute wealth and the actual effect, potentially shifting benefits from vulnerable, short-lived, low-socioeconomic status (SES) individuals to long-lived high-SES ones. Understanding changes in mortality across different sub-groups is, therefore, essential in assessing the consequences of pension policies on socioeconomic inequalities in mortality. Some of the consequences of the recent Danish and Swedish pension policy reforms can be anticipated by forecasting mortality. In this paper, we take advantage of two forecasting methods that use different underlying assumptions on the rate of improvement—changing rates of improvement (Bergeron-Boucher et al., 2024) and constant rates of improvement (Li & Lee, 2005)—to evaluate how different forecast assumptions impact the conclusions. This paper has two objectives: (1) to quantify the differences in contemporary mortality developments after age 50 by SES in Denmark and Sweden, and (2) to assess future impacts of these mortality developments, and consequent changes in statutory retirement age, on inequalities in mortality in Denmark and Sweden using two different forecasting models. These two objectives lay the framework for the two-part structure of the Results and Discussion sections.

## Background

### Population Ageing and Pension Policies in Denmark and Sweden

The Danish pension system is made up of three ‘pillars’: the public national pension and the mandatory savings-based pensions (pillar I), labour market pensions (pillar II), and private pension savings (pillar III). Pillar I caters primarily to people with low incomes to ensure a minimum standard of living. Pillar II accounts for the fact that many individuals maintain coverage of pension income in relation to income as a business asset and thus avoid significant drops in income when withdrawing from the labour market. The private pension schemes in pillar III provide flexibility for individuals to adapt their savings to specific needs and additional coverage. These pillars work in tandem to achieve the three aims of the Danish pension system: to enable savings to maintain a reasonable standard of living, redistribute funds from high-income to low-income individuals, and act as insurance against uncertainty, for example, loss of working capacity before retirement age (Whitta-Jacobsen et al., 2022).

Current Danish legislation mandates a gradual increase in the statutory retirement age as life expectancy increases. Each increase must be supported by a majority in parliament. Increases in the state statutory retirement age are adopted every five years with 15 years’ notice. The state statutory retirement age is determined by calculating life expectancy at age 60 for the total Danish population, plus an assumed increase in life expectancy of 0.6 years during the notice period, subtracted from the assumed state statutory retirement age period of 14.5 years. The state statutory retirement age can be increased by a maximum of one year at a time, and is rounded to the nearest half year by regulation. In 2019, retirement age increased from 65 to 65.5, and in 2022, to 67. In 2040, it is expected to be 70.

Sweden’s pension system is similar to Denmark’s in that it is comprised of three parts: a public pension from the state, an occupational pension from an employer, and savings or assets that an individual may have. The national public pension is based on an individual’s total income in Sweden throughout their working life and is divided into several further parts: income pension, income pension complement, premium pension, and guarantee pension. Most people who have worked in Sweden also receive an occupational pension from their employer. Those eligible for Swedish pensions can apply for income pensions from the month they turn 63, at the earliest, which is due to increase to 64 in 2026. At 66, individuals can receive the guaranteed pension, income pension supplement, and housing supplement, which is due to rise to 67 in 2026. Individuals in Sweden have the right to work to age 68, or later if their employer permits it (Pensions Myndigheten, 2023).

### Social Inequalities in Mortality

SES is typically analysed using three variables—education, employment, and income—either individually or combined (Berkman et al., 2014). The SES gradient in mortality is generally quantified by calculating summary measures, such as life expectancy or age-standardised death rates, which allow for sub-national and international comparisons. For example, these measures can be used to show differences in life expectancy between higher- and lower-SES groups, as in Brønnum-Hansen and Baadsgaard (2012), who found a widening social gap in life expectancy in Denmark over a 25-year period. These measures are effective when summarising inequalities among populations and sub-populations, but hide variation within them. Quantifying lifespan inequalities—the variation in lifespan observed within a population or sub-population—enhances our understanding of the social gradient in mortality (van Raalte & Caswell, 2013; van Raalte et al., 2018). Lifespan inequalities have generally been decreasing over time, suggesting lower variation in lifespans. This has been attributed to mortality reductions at young and middle ages (Vaupel et al., 2011). Additionally, it has been found that preventing deaths before life expectancy contributes to an increase in life expectancy and a reduction in lifespan inequalities (Aburto et al., 2020). While differences in life expectancy between low- and high-SES sub-populations have persisted and, in some cases, increased, many low-SES sub-populations have also experienced increased lifespan inequalities (van Raalte et al., 2018).

Despite robust welfare systems, this pattern is also seen in the Nordic countries (Mackenbach, 2012). Lower socioeconomic groups in the Nordics have experienced little improvements in life expectancy and no reduction in lifespan inequality, suggesting Nordic societies are failing in postponing early deaths to older ages among people of low SES (Brønnum-Hansen et al., 2021). Indexing statutory retirement age to life expectancy is, therefore, potentially harmful to those of low SES. Studies that examine the implications of indexing retirement age to life expectancy reach the same conclusion. Survival to retirement age has been found to be unequal across different socioeconomic groups in Denmark over time, with larger inequalities seen in more recent cohorts of those who have reached retirement age (Strozza et al., 2022). Indexing statutory retirement age to life expectancy has been found to magnify the inequalities experienced by those of low SES and make the financial cost of the system more sensitive to changes in mortality (Alvarez et al., 2021). It also primarily benefits those with a higher level of education, and implementation of a flexible pension scheme to account for health inequalities among occupational groups could reduce inequalities in disability-free life expectancy (Brønnum-Hansen et al., 2017, 2020).

### Forecasting Mortality

The policy of indexing statutory retirement age to life expectancy is recent, and its potential consequences are yet to be observed. However, some trends can be anticipated by forecasting mortality. There are several methods used for forecasting, and no consensus on which model is the best. One of the most used models to forecast mortality is the Lee-Carter (LC) model (Lee & Carter, 1992), which forecasts age-specific death rates log-bilinearly, assuming constant rates of mortality improvement by age. The latter assumption is not adequate in many cases where the age-specific rates of mortality improvements (ASRMI) changing over time. There is a tendency to observe slower rates of improvement at younger ages, but faster rates at older ages, referred to as a rotation (Li et al., 2013; Rau et al., 2008). Despite a mixed performance of the LC model, the model remains popular because of its simplicity; it is a powerful method and limited subjective judgement is required. Many national statistical offices, including those in Denmark and Sweden, use the LC model, or an extension of it, for official national forecasts (Bergeron-Boucher & Kjærgaard, 2022). However, other models have been developed to forecast mortality that account for changing ASRMI that tend to be more accurate, both in terms of life expectancy and lifespan variation (Bergeron-Boucher et al., 2024; Bohk-Ewald & Rau, 2017; Li et al., 2013). These models tend to forecast faster increases in life expectancy by forecasting accelerating mortality decline at older ages. We compare trends using a model from the LC family and another that accounts for changes in ASRMI to assess how both forecasts anticipate inequalities in retirement across SES.

## Data and Methods

### Danish and Swedish Registry Data and Retirement Age

We used data from the Danish and Swedish registries from 1988 to 2019 and 1991 to 2017, respectively, to aggregate individuals based on their demographic and socioeconomic characteristics. The study population includes all residents in Denmark and Sweden during the study period, aged 50 years or more. SES was defined according to individuals’ equivalised disposable family income (Eurostat, 2021). The final amount was obtained by the sum of family members’ individual disposable incomes, divided by the number of equivalent adults living in the household to reflect its size and age composition (more details provided in Appendix). Individuals residing in Denmark and Sweden were then classified based on the quartiles of the income distribution, computed by year, sex, and five-year age groups until age 89, and those 90 + . For this reason, the population is evenly distributed across income quartiles, with each quartile representing 25% of the total population by year, sex, and five-year age group. However, the number of deaths varies by sex and across income quartiles. Table 3 in Appendix shows exposures and deaths by sex and income quartile for both countries and two years.

Statutory retirement ages differ in the two countries. In Denmark, retirement age will increase from 67, set in 2022, to 70 in 2040, with an increase of one year every five years. In Sweden, retirement age increased from 65 to 66 in 2023, and will increase to 67 in 2026. After that, the future retirement age has not been defined. In our study, we assumed an increase of one year every five years (as in Denmark) after 2026 (see Table 4 in Appendix).

### Data Analysis and Measures

We computed period life tables by year, sex, and income for Denmark and Sweden using the aggregated data described in the previous section. Exposures were obtained as the average population alive between two calendar years. Based on the period life tables calculated for each subgroup of the Danish and Swedish populations, we estimated two different groups of measures: those of mortality and longevity, and those of survival to and after retirement age.

#### Measures of Mortality and Longevity

We calculated life expectancy at age 50 to summarise population health over time and the average of the previous 10 years of ASRMI, both by sex and income. The ASRMI ($\rho \left(x,t\right))$ are calculated as:

$$\rho \left( {x,t} \right) = - \log \left( {\frac{{m\left( {x,t + 1} \right)}}{{m\left( {x,t} \right)}}} \right)$$,

where $m(x,t)$ is the death rate at time $t$ and age $x$.

We also quantified lifespan inequalities by calculating e-dagger (e^†^) (Vaupel & Canudas-Romo, 2003) over time by sex and income. This is interpreted as the average remaining life expectancy at death, or, alternatively, the average years of life lost due to death in a population. It is calculated as follows:

$$e^{\dag} = \mathop \smallint \limits_{50}^{\omega } d\left( x \right)e\left( x \right)dx,$$

where $d(x)$ is the life table deaths at age $x$ and $e(x)$ is the remaining life expectancy at age $x$.

We also calculated the modal age at death (*M*).* M* is a measure of longevity, solely influenced by mortality at older ages, and complements $e^{\dag}$, which is influenced by mortality at younger ages. *M* captures the postponement of mortality towards older ages (Canudas-Romo, 2008). We calculated *M* by year, sex, and income with the nonparametric approach put forward by Ouellette and Bourbeau (2011). Because it can be arduous to estimate *M* when non-smoothed or erratic trends are observed, Ouellette and Bourbeau suggest using a P-Spline approach to smooth the age-at-death distribution and find the mode. We applied this method to our analyses.

#### Measures of Survival to and After Retirement Age

To quantify inequalities in access to retirement, we estimated the probability of surviving from age 50 to the statutory retirement age by year, sex, and income. Additionally, we calculated remaining life expectancy at retirement over time to assess the number of years different groups are expected to live in retirement by sex and income.

### Forecast

The official national forecasts for Denmark and Sweden are based on extensions of the Lee-Carter (LC) model (DREAM, 2013; Lee & Carter, 1992; Statistiska centralbyrån, 2018). The LC model extrapolates age-specific death rates log-bilinearly and is one of the most used models to forecast mortality. However, when forecasting multiple populations, the LC model tends to lead to crossover or divergence between populations in the forecast, even when convergence is observed. We found that the LC model did not maintain the SES gradients and crossovers occurred in the forecasts. We then selected a multi-population forecasting model, also known as coherent or augmented common factor (ACF). ACF models assume that mortality between groups is not independent. As all SES groups in Denmark and Sweden benefit from the same welfare systems, the assumption seems justified. We used the Li-Lee (LL) model, which is a coherent extension of the Lee-Carter model (Li & Lee, 2005). The model forecasts a reference population, or a common tendency, with the LC model and then forecasts the deviation between a population and the reference using a stationary time-series model, which does not impose a long-term deviation between the population and the reference. The reference population was the mean death rates across income and sex. Because the life expectancy between sexes has been converging in recent years, reaching similar levels for some income group, we used the total average as the reference, rather than sex-specific average, which allowed us to forecast mortality across both income and sex dimensions and to also avoid crossover between mortality trends for women and men.

To test the sensitivity of the results of our selected forecast model, we also compare the results of the LL model with one that allows for changes in the ASRMI. The LC and LL models are known to underpredict life expectancy, mainly due to their assumption of constant ASRMI in the long term (Bergeron-Boucher & Kjærgaard, 2022). We therefore compare the LL model forecasts with that of a coherent extension of the Mode model. For simplicity, we refer to the coherent extension of the Mode model as the Mode model throughout the text. The Mode model forecasts the age-at-death distribution using the modal age at death in two steps: (1) by forecasting the modal age at death and (2) by forecasting the age-at-death distribution centred around the mode using the compositional data analysis (CoDA) model put forward by Oeppen (2008). Changes in the modal age at death have generally been more linear than changes in the age-specific death rates, providing a strong basis for extrapolation and forecasting. In addition, the model allows us to account for changes in ASRMI, which tends to increase forecast accuracy (Bergeron-Boucher et al., 2024). Evidence shows that, in low-mortality countries, mortality decline is decelerating at younger ages but accelerating at older ages (Li et al., 2013; Rau et al., 2008; Vaupel et al., 2021). This pattern is referred to as a rotation (Li et al., 2013), which the Mode model can account for. Similar to the LL model, the reference population was the mean age-at-death distribution across income and sex. We forecast the modal age at death for the reference population using a random walk with drift and forecast the income group deviation from the reference using a stationary time-series model. The age-at-death distribution around the mode was forecast using a coherent compositional data analysis model (Bergeron-Boucher et al., 2017). Mortality was forecast until 2040.

By comparing these two models, based on different rates of improvement assumption, we were able to derive more robust conclusions of the consequences of statutory retirement age indexation on unequal access to retirement. More details pertaining the two methods are provided in Appendix.

## Results

### Socioeconomic Inequalities in Mortality and Longevity in Denmark and Sweden

**Fig. 1:**
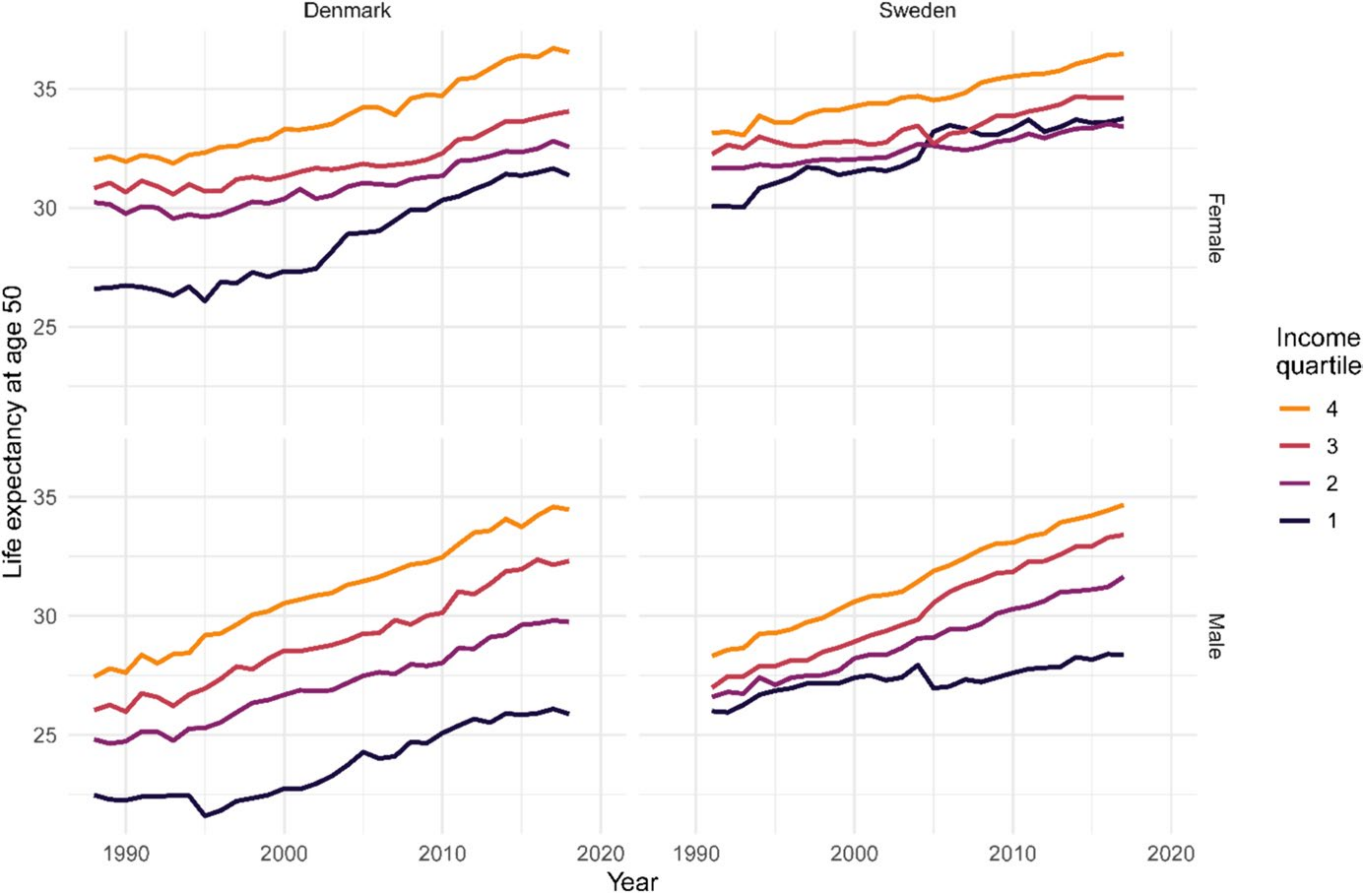
Life expectancy at age 50 by sex and socioeconomic status (income quartile) in Denmark and Sweden. Years 1988–2018 (Denmark) and 1991-2017 (Sweden)

Life expectancy at age 50 differed in the two countries, mainly among those with lower incomes, where Denmark lagged behind Sweden (Fig. 1). Nevertheless, life expectancy followed similar trends in the two countries for all income groups and for both sexes over the course of the study period. For men, progress in life expectancy has been slower for the lowest income group, leading to increasing inequalities over time. From 1988 to 2018 for Denmark and 1991 to 2017 for Sweden, life expectancy increased by 3.4 and 2.3 years for the first (lowest) income quartile, 4.9 and 5.0 years for the second, 6.3 and 6.4 years for the third, and 7.0 and 6.4 years fourth (highest) income quartile, respectively, in Denmark and Sweden. For women, life expectancy increases have been smaller for the second- and third-income groups: 2.3 and 3.2 years in Denmark, and 1.8 and 2.4 years in Sweden, respectively. On the other hand, life expectancy increases were greater and roughly equal between the first and fourth groups, with an increase of 4.8 years in the first and 4.5 years in the fourth group in Denmark. In Sweden, the corresponding increases were 3.7 and 3.3 years. Inequalities reduced across the first three income groups for women and increased for men.

**Fig. 2:**
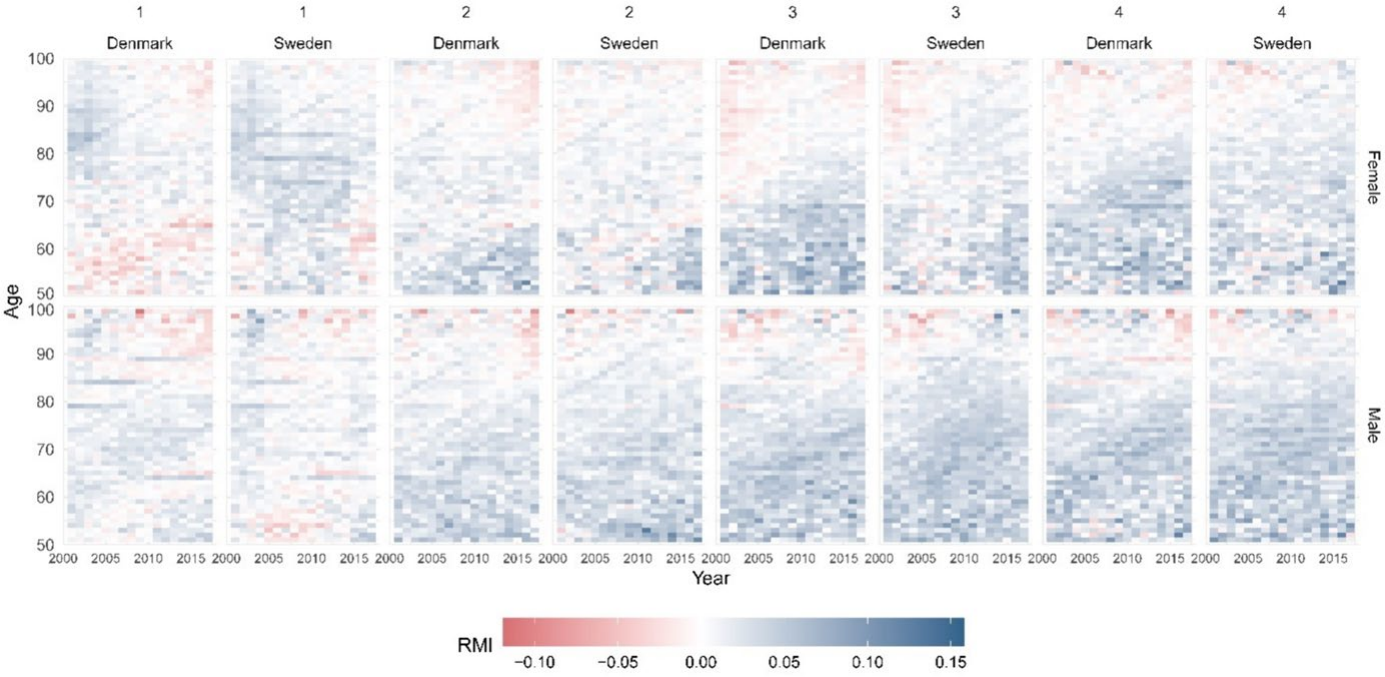
Age-specific Rates of Mortality Improvements (ASRMI) by sex and socioeconomic status (income quartile) in Denmark and Sweden. Years 1998–2018 (Denmark) and 1998–2017 (Sweden)

To better understand these developments, we looked at the ASRMI, i.e. how fast mortality has been changing by age for each income group and by sex. Figure 2 shows a clear cohort effect for the second to the fourth income groups, which is particularly strong in Denmark and for women in both countries, with worsening mortality for the cohorts born before the Second World War. A similar cohort effect was observed for men, but for older generations, while a mortality decline for younger generations was observed at most ages for all the income quartiles except the first.

A surprising result is the absence of this cohort effect for women from the first income quartile (lowest) in both countries and a smaller cohort effect for men. Mortality has decreased between ages 70 and 95 in Denmark and 65 and 95 in Sweden since the early 2000s for women in this group. However, a new cohort effect appears for both sexes in this group in Denmark and for men in Sweden; individuals born in the 1950s had worse mortality than the previous and following cohorts in the first income quartile (Fig. 2).

The mortality progress at older ages for the lowest income groups can be offset by mortality worsening at younger ages, both slowing down the progress in life expectancy and increasing lifespan inequality (Table 1). Indeed, there was an increase in lifespan inequalities for the first income quartile (lowest income group) in both countries. There was increased uncertainty for individuals in this group regarding the time of death and the probability of reaching old ages, including retirement age. This increase was more pronounced in Denmark, where the overall levels of lifespan inequalities are higher than in Sweden. All other income groups, in both countries, were characterised by a sustained decrease in lifespan inequalities over time (see Fig. 5 in Appendix).

**Table 1:** Lifespan inequality (e†) and modal age at death (*M*) by sex and socioeconomic status (income quartile) in Denmark and Sweden. Years 1991 and 2017

Income quartile	Female				Male			
	Denmark		Sweden		Denmark		Sweden	
	1991	2017	1991	2017	1991	2017	1991	2017
*Lifespan inequality at age 50*								
1	8.66	9.93	8.55	8.75	9.20	10.60	9.46	9.55
2	10.15	8.80	8.86	8.15	9.86	8.78	9.17	8.00
3	10.16	7.58	8.79	7.83	9.86	7.86	8.93	7.63
4	9.42	7.46	8.34	7.48	9.22	7.50	8.57	7.32
*Modal age at death*								
1	81.5	88.1	84.2	89.3	75.0	83.8	79.9	85.3
2	87.4	87.4	86.8	87.6	76.8	84.6	80.6	85.7
3	87.0	86.9	86.3	88.4	78.9	85.5	80.0	86.7
4	87.6	90.2	87.4	89.4	81.7	88.5	81.7	88.0

The levels of the modal age at death have been converging over time among the three lowest income groups, especially among women, suggesting more similar longevity (see Fig. 6 in Appendix). As reported in Table 1, there was a postponement of mortality, i.e. a shift of the lifespan distribution towards older ages in Denmark and Sweden for both sexes and all income groups, with the exceptions of the second and third income groups for women in Denmark. Knowing that the modal age at death is solely influenced by mortality at older ages, this trend is explained by negative improvements or no improvements in mortality above age 80 observed for those groups during the study period (see Fig. 2).

These results suggest that the difference in life expectancy, as shown in Fig. 2, is most likely driven by differences in mortality at younger ages. Mortality at older ages is less different across groups, except among individuals from the highest income group, who also have a clear survival advantage at older ages.

### Survival to and After Retirement Age in Denmark and Sweden: Past, Present, and Future

**Fig. 3:**
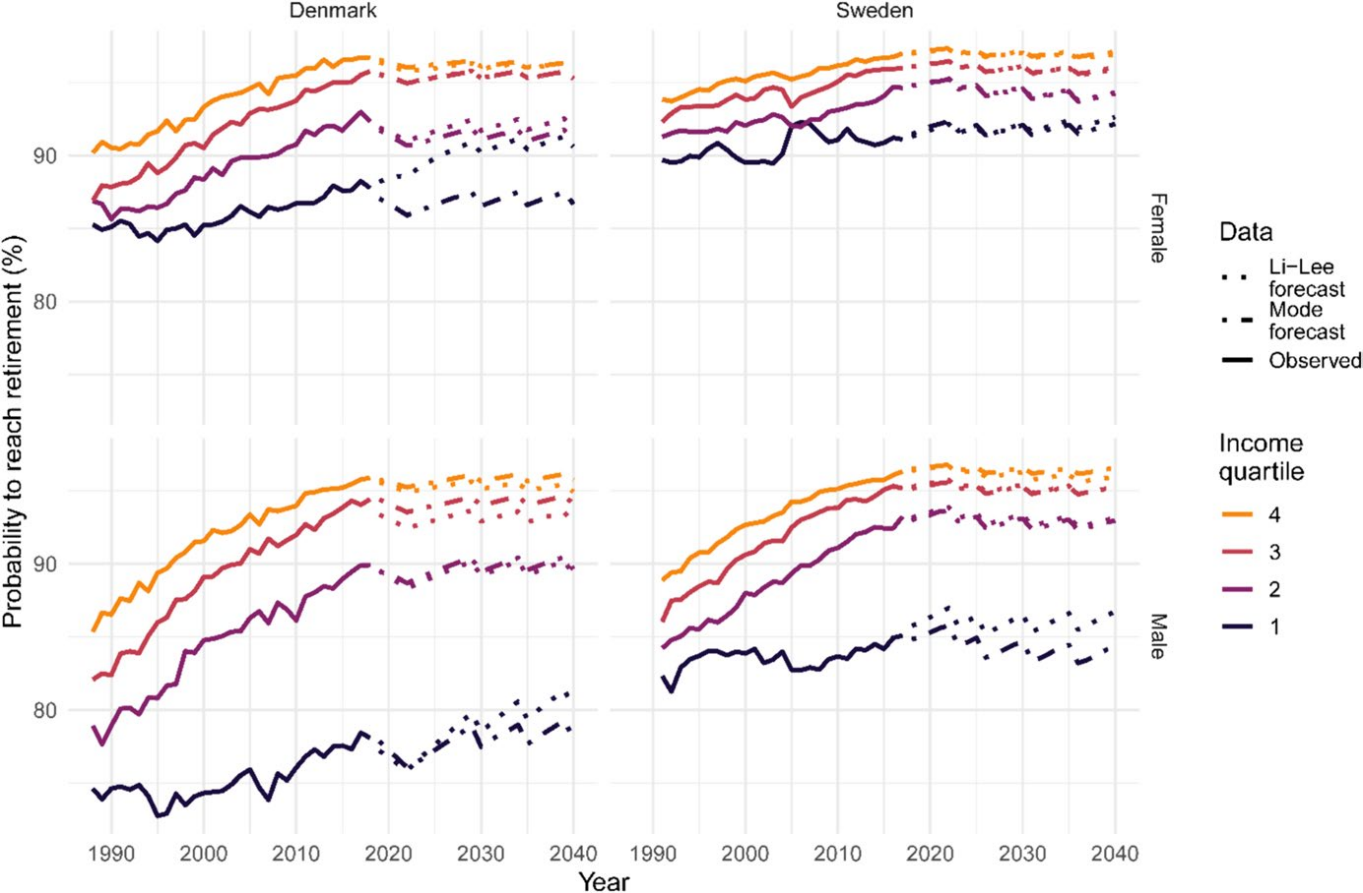
Probability of surviving to retirement age by sex and income quartile in Denmark and Sweden. Observed data: years 1988–2018 (Denmark) and 1991–2017 (Sweden). Two models are used to forecast mortality: Li-Lee and Mode: years 2019–2040 (Denmark) and 2018–2040 (Sweden)

How do these mortality differences translate into access to retirement and time spent in retirement? How does indexing statutory retirement age to life expectancy affect different income groups? Figure 3 shows the observed probability of reaching retirement (age 65) in Denmark from 1988 and Sweden from 1991 over the observed period, and the forecast, under the current indexation scheme. Indexation does slow down, and even level off, the increase in the probability of reaching retirement that was observed in the past. Generally, Sweden has higher survival to retirement age for all income groups. Still, social inequalities in survival to retirement age in Denmark and Sweden increased during the study period due to a smaller improvement in survival for the lowest income group. The two highest income groups present similar levels of survival to retirement age in both countries, but in Sweden, we observed higher survival for the two lowest income groups compared to Denmark. The forecast was performed with the LL and Mode models. Both models forecast little difference in terms of access to retirement over time for the three highest income groups in both countries. However, the LL model forecast an increase in access to retirement for the lowest income group in Denmark and among men in Sweden, while the Mode model predicted little change.

**Fig. 4:**
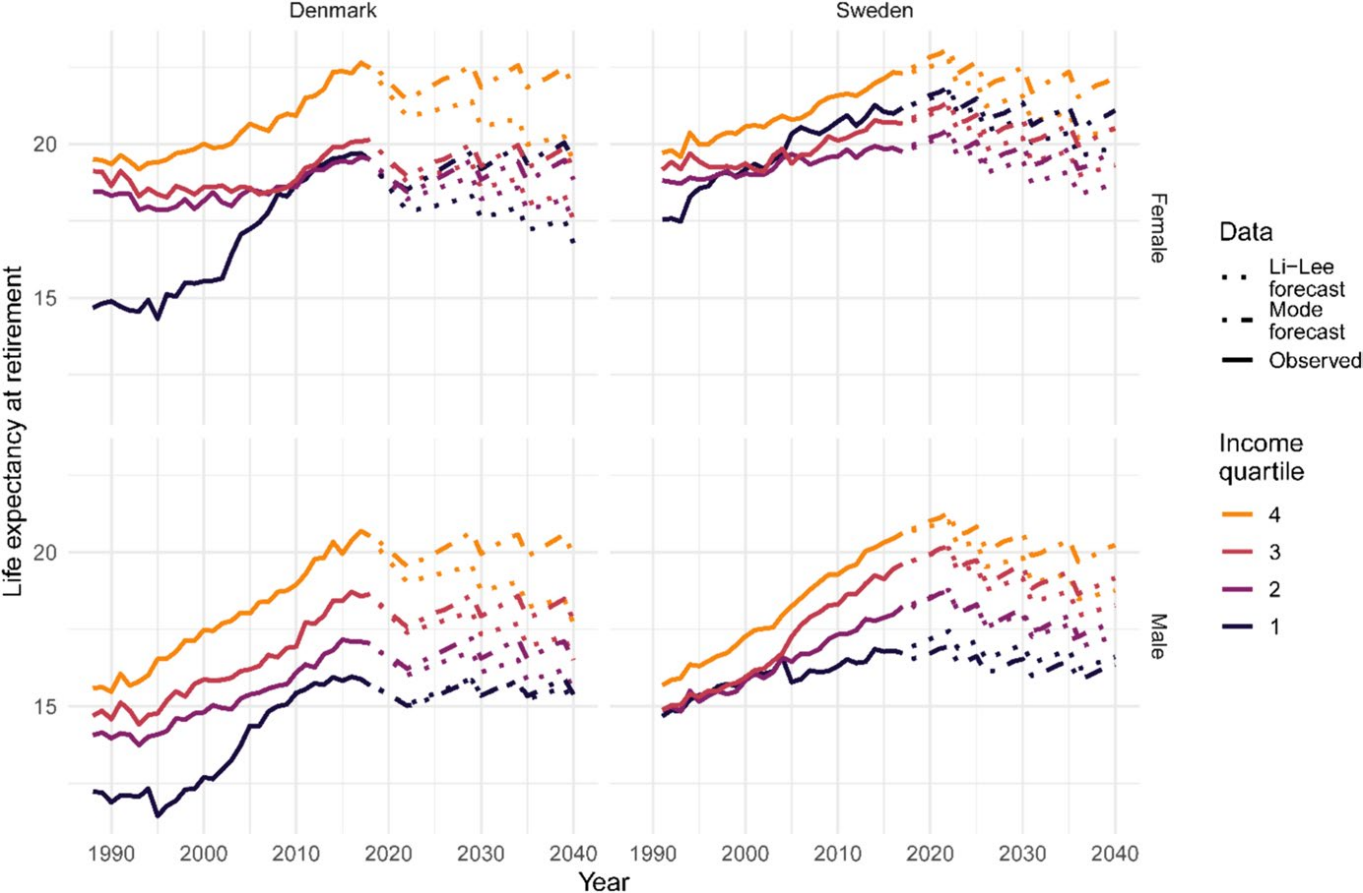
Life expectancy at retirement by sex and income quartile in Denmark and Sweden. Observed data: years 1988–2018 (Denmark) and 1991-2017 (Sweden). Two models are used to forecast mortality: Li-Lee and Mode: years 2019–2040 (Denmark) and 2018-2040 (Sweden)

**Fig. 5:**
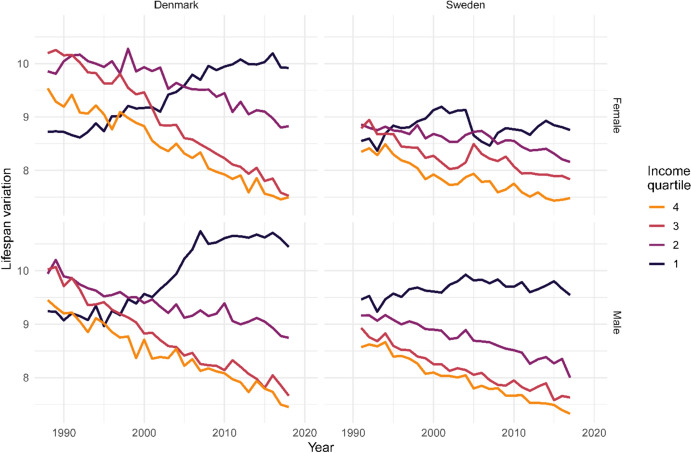
Lifespan variation measured through e† by sex and income quartile in Denmark and Sweden. Years 1988–2018 (Denmark) and 1991–2017 (Sweden)

**Fig. 6:**
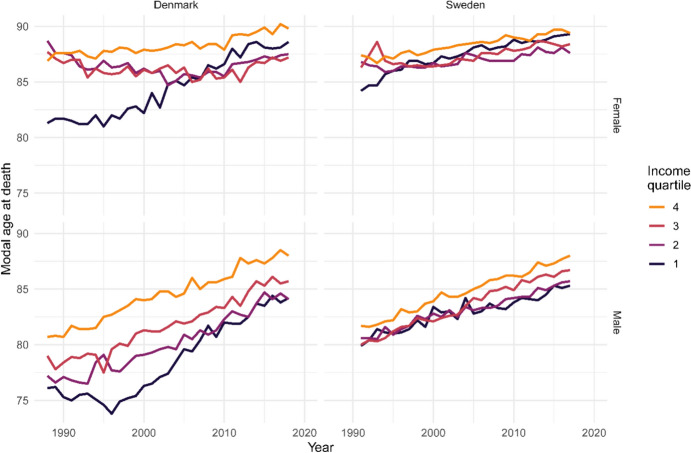
Modal age at death by sex and income quartile in Denmark and Sweden. Years 1988–2018 (Denmark) and 1991–2017 (Sweden)

By indexing the statutory retirement age, one of the goals of the Danish and Swedish governments is to reduce the time that individuals are expected to spend in retirement. Figure 4 shows life expectancy at retirement (*e*_R_) observed between 1988 and 2018, and forecast, with the indexation of the statutory retirement age. In Denmark, social inequalities in *e*_R_ reduced over the observed period while they remained stable at a low level in Sweden. The LL model forecasts a decrease in *e*_R_ over time for all income groups in both countries, while the Mode model forecasts a (somewhat) constant *e*_R_ over time.

**Fig. 7:**
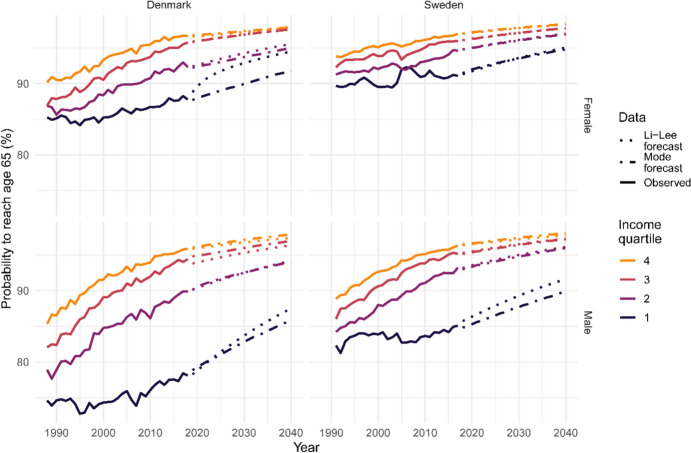
Probability of surviving to age 65 by sex and income quartile in Denmark and Sweden. Observed data: years 1988–2018 (Denmark) and 1991–2017 (Sweden). Two models are used to forecast mortality: Li-Lee and Mode: years 2019–2040 (Denmark) and 2018-2040 (Sweden)

**Fig. 8:**
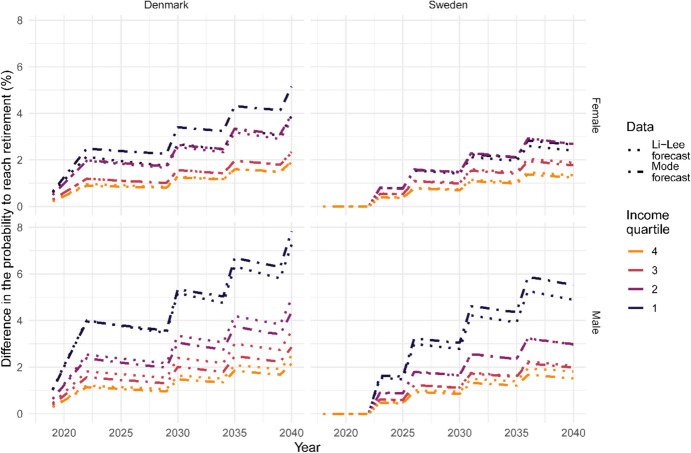
Differences in the probability of surviving to retirement age by sex and income quartile in Denmark and Sweden between two scenarios: no-indexation and indexation of retirement age. Two models are used to forecast mortality: Li-Lee and Mode. Years 2019–2040 (Denmark) and 2018–2040 (Sweden)

Table 2 shows the difference in the probabilities of reaching retirement if the statutory retirement age remains at age 65 (see Fig. 7 for the time series), and if it is indexed. Indexing the statutory retirement age with life expectancy disproportionally affects the lowest income group in terms of access to retirement, both in Denmark and Sweden, especially among men. The difference in Denmark is greater than in Sweden; however, this is partially explained by the last increase in statutory retirement age expected in 2040 for Denmark that does not occur in Sweden (see Table 4 in Appendix). By 2040, the decrease in the probability of reaching retirement created by the indexation for the lowest income group will be over two times higher than that of the highest income group, with both models for both countries (see Fig. 8 in Appendix for the time series).

**Table 2:** Differences in the probability of surviving to retirement age and life expectancy at retirement by sex and income quartile in Denmark and Sweden for two scenarios: no indexation and indexation of retirement age

Income quartile	Female				Male			
	Denmark		Sweden		Denmark		Sweden	
	Li-Lee	Mode	Li-Lee	Mode	Li-Lee	Mode	Li-Lee	Mode
*Difference in probability of surviving to retirement age (%)*								
1	5.15	3.89	2.66	2.40	7.83	7.26	5.55	4.89
2	3.94	3.72	2.70	2.68	4.36	4.94	2.99	2.98
3	2.32	2.34	1.77	1.88	2.86	3.54	1.99	2.06
4	1.92	1.90	1.24	1.34	2.15	2.50	1.52	1.81
*Difference in life expectancy at retirement age (years)*								
1	3.77	4.21	3.35	3.45	3.37	3.55	2.87	3.01
2	4.12	4.23	3.4	3.43	4.13	4.07	3.39	3.41
3	4.49	4.52	3.6	3.59	4.41	4.31	3.57	3.57
4	4.53	4.58	3.7	3.69	4.51	4.49	3.66	3.62

Two models are used to forecast mortality: Li-Lee and Mode. Year 2040

**Fig. 9:**
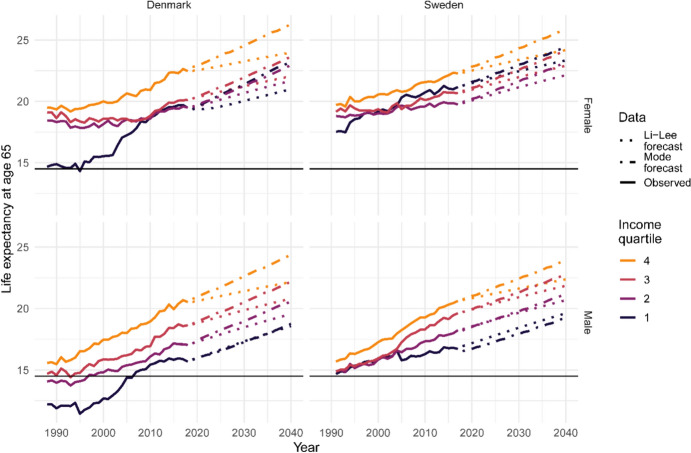
Differences in life expectancy at age 65 by sex and income quartile in Denmark and Sweden between two scenarios: no-indexation and indexation of retirement age. Observed data: years 1988–2018 (Denmark) and 1991–2017 (Sweden). Two models are used to forecast mortality: Li-Lee and Mode. Years 2019–2040 (Denmark) and 2018–2040 (Sweden)

**Fig. 10:**
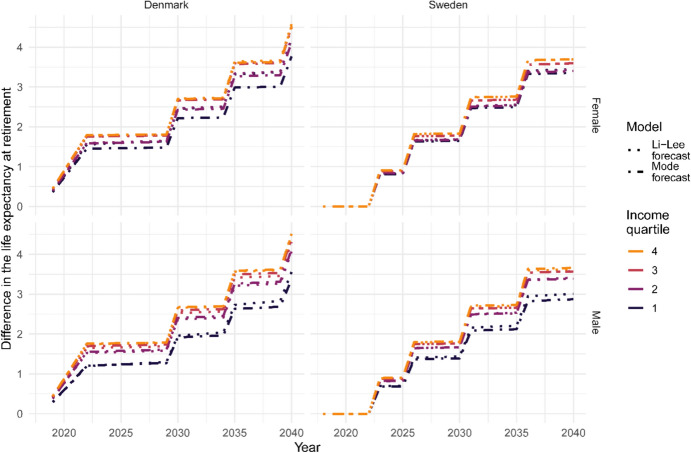
Differences in life expectancy at retirement by sex and income quartile in Denmark and Sweden between two scenarios: no-indexation and indexation of retirement age. Two models are used to forecast mortality: Li-Lee and Mode. Years 2019–2040﻿ (Denmark) and 2018–2040 (Sweden)

Table 2 also shows the number of years in retirement lost by indexing the statutory retirement age in Denmark and Sweden. It shows the difference in life expectancy between age 65 (retirement age without indexation, see Fig. 9 for the time series) and *e*_R_ (life expectancy at retirement with indexation). Both forecast models provide similar results: indexing statutory retirement age has a greater impact on the highest socioeconomic group. Indexing statutory retirement age will reduce the number of years spent in retirement in Denmark by around 4.5 years for the highest income group and by 3.5 years for the lowest income group for males by 2040, and by 4.5 and 4 years for females in Denmark, on average with both models. In Sweden, with the last assumed increase occurring in 2036 and no increase in statutory retirement age expected in 2040, the differences are about one year smaller than in Denmark (see Fig. 10 in Appendix for the time series).

## Discussion

With ageing populations threatening the sustainability of pension systems, Denmark, Sweden, and many other European countries have instigated a system whereby statutory retirement age is linked to life expectancy through an indexation policy. For instance, in Denmark, with approval from parliament, statutory retirement age is set to rise in steps to age 70 in 2040. This is done to account for improvements in mortality. However, improvements in mortality, i.e. increases in life expectancy, are calculated on the population level and do not account for inequalities within the total population. Such an approach may inadvertently exacerbate existing disparities, even in countries with flexible schemes and options for early exit, as it fails to account for differences in life expectancy across socioeconomic groups, potentially misaligning the intended redistribution goals of the pension system (Alvarez et al., 2021; Strozza et al., 2022). The basis for this paper was twofold: to quantify mortality developments by SES in Denmark and Sweden, and to forecast how these mortality developments and consequent changes in statutory retirement age will impact social inequalities in survival to and after retirement age in Denmark.

### Mortality Developments by SES in Denmark and Sweden (Objective 1)

Lower socioeconomic groups have lower life expectancies and greater lifespan inequalities than those of higher socioeconomic groups. This result is consistent across education, income, and occupation classes when used to define socioeconomic groups (Strozza et al., 2022; van Raalte et al., 2018). Over the past decades, social inequalities in mortality have been widening in Denmark, Sweden, as well as in other European countries (Brønnum-Hansen & Baadsgaard, 2012; Huisman et al., 2004; Mackenbach, 2017; Mackenbach et al., 2003). This trend has mostly been studied in terms of education and income groups, and the results obtained with these two indicators have different interpretations. When education is used as a proxy of SES, widening mortality inequalities over time are often attributed to the selection of those who are in the most disadvantaged group, and to the difference in what it means to be less or more educated in different time periods (Brønnum-Hansen & Baadsgaard, 2012). On the other hand, when income is used as a proxy for SES, widening mortality inequalities over time are partially attributed to the change in the income distribution of a population (Brønnum-Hansen et al., 2021). In this paper, we investigated mortality and longevity developments in Denmark and Sweden by income to quantify social inequalities in mortality in two Nordic countries, and therefore, we must make the necessary considerations. We used income as proxy for SES because of its availability throughout the study period and across age groups. Education data are missing for the older generations for a large part of the study period, which would negatively affect the reliability of our estimates and forecast. Overall, we observed an increase in life expectancy at age 50 in Denmark and Sweden for all income groups and both sexes, in line with research that concludes that life expectancies in countries with the longest lifespans are increasing. However, we found that men in the lowest SES groups experienced increased lifespan inequalities over the study period. This is consistent with research that finds widening inequalities in mortality by SES in the Nordic region (Brønnum-Hansen & Baadsgaard, 2012; Brønnum-Hansen et al., 2021; Mackenbach, 2012; Strozza et al., 2022). In particular, our results are in line with those of Strozza et al. (2022) who focus on cohort survival between ages 50 and 70 by socioeconomic status. There is a breadth of literature on the link between cohort and period mortality that shows that period mortality can be also seen as lagged cohort mortality. What period mortality does not capture is improvements in mortality that the so-called synthetic cohort would otherwise experience throughout their lives. Our period estimates are comparable to the cohort estimates in Strozza et al. (2022), with the magnitude of the inequality across income groups being slightly larger in the cohort estimates. We attribute this difference to the different definition of income quartiles in the period and cohort settings. The period estimates are influenced by dynamics of the income distribution across cohorts, calculated by five-year age groups. This signals that improvements in mortality are not homogeneous within the population, and lays the groundwork for a deeper investigation into the source of such inequalities.

For this reason, we looked at the ASRMI to determine how fast mortality has been changing over time, by age, across socioeconomic groups. While clear cohort effects were observed for some income groups, the absence of a cohort effect in the lowest income quartile was noteworthy, particularly with regard to worsening mortality for the cohorts born after the Second World War. It has been shown that women born between 1915 and 1945 had particularly high mortality, likely due to a high prevalence of smoking among women in these cohorts (Lindahl-Jacobsen et al., 2006, 2016). Similarly, Aburto et al. (2018) find that since 1960, Danish improvements in life expectancy and lifespan inequality were halted by smoking-related mortality in the cohorts born between 1919 and 1939. Kallestrup‐Lamb et al. (2020) find very similar results to ours in their analysis on cause-specific mortality by socioeconomic groups in Denmark. They conclude that a long period of stagnation in mortality was observed among Danish women between 1985 and 1995 in the mid-socioeconomic groups, caused by an increase in mortality from cancer and other causes. On the other hand, they also find that mortality among the lowest socioeconomic group improved due to a reduction in cardiovascular and cerebrovascular disease mortality. Additionally, given that smoking-related mortality and other health-risk behaviours tend impact mortality among low-SES individuals more than high-SES ones, one potential explanation could be a selection effect. Individuals who survived to higher ages are selected individuals that tend to be healthier and less frail. A new cohort effect appears for individuals from the first income quartile (lowest) in Denmark and, to some extent, among Swedish men. Individuals born in the 1950s had higher mortality than the previous and following cohorts in the first income quartile. It is possible that this new cohort effect is due to an increase in smoking prevalence for individuals of the lowest SES group in these cohorts, but other factors could also explain this trend (Osler et al., 2001). Interpreting cohort mortality patterns from period data might be problematic, especially in contexts where period data are obtained from censuses (Cairns et al., 2016). In this study, we used registry data (i.e. data registered on a continuous basis, with all individual information linked via a personal identifier), which are only marginally affected by misregistration. One potential source of bias for our mortality estimates is the over-coverage of migrants who return to their home countries after retirement without deregistering from population registries. Statistics Denmark reports no evidence of data artefacts either by under-coverage (when migrants fail to register upon arrival) or over-coverage, in Danish registry data (Callaway et al., 2024; Danmarks Statistik, 2020). In Sweden, however, there is evidence of over-coverage among migrants up to age 40, but this has been reported to have a small impact on mortality estimates at ages 40–75 (Callaway et al., 2024; Mussino et al., 2023).

Additional information is provided by the analysis of lifespan inequality across socioeconomic groups, as the information on the variability of ages at death is crucial for effective policy planning (Alvarez et al., 2021). Generally, as life expectancy increases, lifespan inequality decreases (Aburto et al., 2020); however, we find that lifespan inequality increased for the lowest socioeconomic groups in both Denmark and Sweden. This result is in line with previous research that finds that the Nordic countries do not postpone early deaths among those with low SES (Brønnum-Hansen et al., 2021).

Life expectancy and lifespan variation provide partial information on longevity extension, which is best measured by the modal age at death. The modal age at death captures the most common lifespan and indicates the timing of death (Horiuchi et al., 2013). We observe an overall increase in the modal age at death among men in Denmark and Sweden, with those in the lowest socioeconomic group experiencing a greater improvement than that the others. Among women in both countries, the lowest socioeconomic group experienced the biggest improvement, surpassing the two mid-socioeconomic groups in terms of longevity. This result is, again, a consequence of the development of the ASRMI at older ages, as previously discussed. The modal age at death is an indicator that is solely influenced by mortality reduction at older ages (Canudas-Romo, 2008, 2010; Horiuchi et al., 2013). It is worth emphasising that the analysis is based on period data. Thus, as previously highlighted, these results do not capture cohort dynamics. For each life table, age-specific mortality rates are assumed to remain constant during the life course and we assume there is no transition across income quartiles. In other words, each individual is assumed to stay in the same income quartile for the remainder of their lives, which may not reflect the reality. That said, because of the structure of the Danish and Swedish pension systems, we assume transition across income quartiles to be minimal. Furthermore, although we used an equivalised indicator of family disposable income, the measurement might be slightly different in Denmark and Sweden. In Sweden, a change in the calculation of equivalised disposable income was registered in 2004, as acknowledged in Appendix. In our analysis, we did not account for the health conditions of our study population. In fast-ageing societies, health, and disability in particular, becomes a crucial focus point for pension policy makers, among other key stakeholders. For instance, it determines the ability of individuals to participate in the labour market. For this reason, and due to the high correlation between health and mortality, using individual income to generate quartiles might reflect individuals’ health conditions. In their last year of life, individuals are likely losing their ability to work or, if they have not, they might only work for a portion of the year. To partly overcome this issue, we have lagged our income variable, so that it does not reflect the income situation of the year the individual died. However, with this strategy we are not able to completely eliminate selection bias in the income groups based on health status.

### Impact of the Indexation Policy on Mortality Inequalities at Retirement (Objective 2)

Looking at the future of social inequalities in mortality allows for better policy planning and an evaluation of the indexation policy in place in Denmark and Sweden. For this purpose, we forecast mortality with two different methods: a coherent extension of the current model employed in Denmark and Sweden (LL), and a coherent extension of the Mode model developed by Bergeron-Boucher et al. (2024). We use these models to forecast the probability of surviving to retirement age and remaining life expectancy in retirement. In the indexation scenario, both models predict that the probability of surviving to retirement age will not change over time for the three highest income groups. However, the LL forecasts a higher probability of survival to retirement age for the lowest income group, while the Mode model predicts little change for this group. This result is mainly due to the assumption behind both models. The LL model forecasts a faster mortality decrease (ASRMI) at younger ages than the Mode model, resulting in a faster decline in lifespan inequalities and an increased probability of reaching retirement. The Mode model, due to its rotation, forecasts a gradual slowdown in the ASRMI at younger ages (Bergeron-Boucher et al., 2024). In terms of time spent in retirement, the LL model forecasts a decrease in remaining life expectancy at retirement while the Mode model forecasts no changes until 2040. These differences can also be explained by different forecast assumptions for ASRMI. With the LL model, the ASRMI at older ages are assumed to remain constant. As the ASRMI are usually lower at older ages, the model forecasts a gradual slowdown in life expectancy as more and more people reach older ages. With the LL model, the statutory retirement age increases faster than life expectancy, resulting in a decrease in remaining life expectancy at retirement. However, the Mode model forecasts an acceleration in the ASRMI at older ages. Life expectancy is forecast to increase at a more similar pace to the statutory retirement age, leading to a constant remaining life expectancy at retirement.

These results are based on two forecasts using distinct assumptions regarding the pace of decline in mortality by age. Which model is the most accurate is a difficult question and has been shown to depend on the population and period of interest. For Denmark, there was an accelerated decline in mortality in recent years, mainly due to a cohort effect. Coherent models using other countries as the reference (which generally produce higher, but constant ASRMI) or models using changing ASRMI over time have been shown to produce more accurate forecasts for Denmark (Bergeron-Boucher & Kjærgaard, 2022; Bergeron-Boucher et al., 2020, 2024; Bohk-Ewald & Rau, 2017). For Sweden, the national ASRMI at older ages have been somewhat constant for females, and research shows that forecast models from the LC family perform well in Sweden (Bergeron-Boucher & Kjærgaard, 2022). However, there is always uncertainty regarding the future, and which of the two models is the most likely remains an open question.

Nevertheless, while the forecast probability of reaching retirement and the number of years in retirement vary between forecast models, both models agree that indexing the statutory retirement age will reduce inequalities in terms of years lived in retirement, but will increase inequalities in terms of access to retirement. Using the independent version of both models leads to the same conclusions (see Table 5 in Appendix). These results are obtained by comparing the forecasts in the two scenarios: one in which there is no indexation (retirement age is constant at age 65) and one in which the indexation policy is in place. As the probability of surviving to age 65 would improve more among the low-SES group without indexation, in absolute terms, implementing indexation would disproportionally affect this group. In essence, raising the statutory retirement age may mean that more individuals in the lowest SES bracket might not live to experience retirement. This effect essentially translates to a higher ‘cost’ of indexation for those who are most economically vulnerable. One of the goals of the pension systems in both Denmark and Sweden is to redistribute funds from high-income to low-income individuals. This is achieved, for example, by allowing individuals to accrue pension rights through various means, including caring for younger children, days of sickness and unemployment, and disability. It is unlikely that the indexation of the pension system in both Denmark and Sweden fully meets this goal of redistribution. Low-income individuals contributing to pension funds are less likely to see the return of their investment than individuals from high-income groups. Increasing retirement age will slow down or halt the convergence between income groups compared to having a fixed retirement age of 65. Nevertheless, indexation will most likely reduce or at least stabilise the currently increasing number of years spent in retirement, especially for the high-income groups, and may further reduce the gap in terms of time spent in retirement between income groups.

## Data Availability

Access to individual-level data is restricted by Danish law. Danish registry data can be accessed with authorisation by Statistics Denmark.

## Appendix

### Equivalised Disposable Family Income

In Denmark, disposable income includes tax-free income plus imputed rent minus interest expenses, taxes, etc. For homeowners, an estimation of their potential rent is added to the total family income. The equivalised disposable family income is obtained as the sum of family members’ individual disposable incomes divided by the number of equivalent adults living in the household (Eurostat, 2021). In order to obtain the number of equivalent adults, all members of the household are weighted by a factor of 1 for the first adult, 0.5 for the second adult and each subsequent person aged 14 or older, and 0.3 to each child under the age of 14.

In Sweden, disposable income is defined as the total incomes after deducting interest expenses, taxes, and other liabilities. The equivalised disposable family income is obtained as the sum of family members’ individual disposable incomes divided by the number of equivalent adults living in the household. In order to obtain the number of equivalent adults, all members of the household are weighted by a factor. From 1991 to 2003, the individual weighting factor applied to family members was 1.16 for the first adult, 0.76 for the second adult, and 0.96 for each additional adult. Children were weighted differently based on age: 0.56 for children age aged 0–3, 0.66 for children aged 4–10, and 0.76 for children aged 11–17. In 2004, these consumption weights were adjusted to align more closely with the common definition employed in most EU countries. The new weightings were 1.0 for the first adult, 0.51 for the second adult, and 0.6 for any additional adult. The first child aged 0–19 was assigned a weighting of 0.52, while any additional children aged 0–19 were assigned a weighting of 0.42. Negative values for individual disposable income and negative value for individualised disposable income were removed from the analyses.

### Methodology

To ensure coherent forecast between populations defined by sex and socioeconomic status, we use the coherent extension of the Lee-Carter model (Lee & Carter, 1992; Li & Lee, 2005) and developed a coherent extension of the Mode model (Bergeron-Boucher et al., 2024).

The coherent Lee-Carter model is defined as:

$$\ln \left( {m_{x,t}^{s,i} } \right) = a_{x}^{s,i} + \beta_{x}^{{}} \kappa_{t}^{{}} + b_{x}^{s,i} k_{t}^{s,i} + \varepsilon_{x,t}^{s,i}$$

where $m_{x,t}^{s,i}$ are the death rates at age *x*, time *t*, sex *s*, and income group *i*, and $a_{x}^{s,i}$ is the age-specific mean of the logged death rates by sex and income group. The $\beta_{x}^{{}} \kappa_{t}^{{}}$ is the common pattern across populations, estimated from a standard Lee-Carter model applied to the mean logged death rates across populations (sex and income group). The $b_{x}^{s,i} k_{t}^{s,i}$ captures the deviation from the common trend for sex *s* and income group *i*. The $\kappa_{t}^{{}}$ and $k_{t}^{s,i}$ are time indexes capturing the change in $\ln \left( {m_{x,t}^{s,i} } \right)$ over time and deviation from the common trend over time. The $\beta_{x}^{{}}$ and $b_{x}^{s,i}$ are age-specific sensitivity to the time indexes. The $\kappa_{t}^{{}}$ is forecast with a random walk with drift and $k_{t}^{s,i}$ with stationary time series.

The coherent extension of the Mode model comprises three steps:

1. Smoothing mortality with the P-Spline approach (Camarda, 2012).
2. From the smoothed age-at-death distributions, we estimated the modal age at death (*M*) for the mean age-at-death distribution and the population-specific modal age at death $(M^{s,i} )$. *M* was forecast with a random walk with drift, and we forecast the difference between *M* and $M^{s,i}$ with a stationary time-series model.
3. The age-at-death distribution centred around the mode by population ($$d_{x - M,t}^{s,i} )$$ was forecast with a CoDa-Coherent model (Bergeron-Boucher et al., 2017):
  $${\text{clr}}\left( {d_{x - M,t}^{s,i} } \right) = a_{x - M}^{s,i} + \beta_{x - M} \kappa_{t} + b_{x - M}^{s,i} k_{t}^{s,i} + \varepsilon_{x,t}^{s,i}$$

where clr is a centred-log transformation. The rest of the parameters have similar interpretations as those in the coherent-LC model (Figs. 5, 6, 7, 8, 9 and 10).  The $\kappa_{t}$ is forecast with a natural cubic smoothing spline and $k_{t}^{s,i}$ with stationary time series (Tables 3,4 and 5).

**Table 3:** Exposures and deaths above age 50 by sex and income quartile in Denmark and Sweden. Years 1991 and 2017

Income quartile	Female				Male			
	Denmark		Sweden		Denmark		Sweden	
	Exposures	Deaths	Exposures	Deaths	Exposures	Deaths	Exposures	Deaths
*1991*								
1	211,233	10,180	385,830	13,969	173,971	8834	322,705	12,354
2	211,231	6105	382,921	10,789	173,969	6471	324,836	11,534
3	211,229	5520	382,586	9872	173,967	5577	325,076	11,370
4	211,227	5123	378,475	9133	173,963	4848	321,704	9919
*2017*								
1	277,563	7020	459,506	11,193	254,499	8581	418,480	13,239
2	277,577	6748	460,365	12,560	254,519	6621	417,340	10,599
3	277,574	6529	458,761	11,103	254,515	5290	416,313	8713
4	277,572	4569	457,790	9098	254,512	3876	415,206	7522

**Table 4:** Indexed statutory retirement age in Denmark and Sweden as used in the analysis. Years 2019–2040

Year	Denmark	Sweden
2019	65.5	65
2020	66	65
2021	66.5	65
2022	67	65
2023	67	66
2024	67	66
2025	67	66
2026	67	67
2027	67	67
2028	67	67
2029	67	67
2030	68	67
2031	68	68
2032	68	68
2033	68	68
2034	68	68
2035	69	68
2036	69	69
2037	69	69
2038	69	69
2039	69	69
2040	70	69

In Sweden, we assumed a one-year increase in statutory retirement age every five years from 2027

**Table 5:** Differences in the probability of surviving to retirement age and life expectancy at retirement age by sex and income quartile in Denmark and Sweden between two scenarios: no-indexation and indexation of retirement age

Income quartile	Female				Male			
	Denmark		Sweden		Denmark		Sweden	
	Lee-Carter	Mode	Lee-Carter	Mode	Lee-Carter	Mode	Lee-Carter	Mode
*Difference in probability of surviving to retirement age (%)*								
1	5.52	4.51	2.91	3.05	9.21	14.65	7.14	6.56
2	4.58	4.56	4.06	4.24	7.44	5.16	2.62	2.31
3	2.07	4.78	1.75	2.82	2.46	2.69	1.38	1.78
4	1.48	1.14	1.28	1.75	1.66	1.81	1.03	1.18
*Difference in life expectancy at retirement age (years)*								
1	3.67	3.91	3.28	3.23	2.96	2.27	2.61	2.65
2	4.10	4.17	3.20	3.21	3.69	3.96	3.49	3.51
3	4.61	4.09	3.64	3.40	4.53	4.43	3.70	3.58
4	4.67	4.74	3.71	3.61	4.65	4.57	3.77	3.71

Two models are used to forecast mortality in their independent version: Li-Carter and Mode. Years 2019–2040

## Code Availability

R code is available upon request.
